# Differential Effects of Early- and Late-Life Access to Carotenoids on Adult Immune Function and Ornamentation in Mallard Ducks (*Anas platyrhynchos*)

**DOI:** 10.1371/journal.pone.0038043

**Published:** 2012-05-30

**Authors:** Michael W. Butler, Kevin J. McGraw

**Affiliations:** School of Life Sciences, Arizona State University, Tempe, Arizona, United States of America; Columbia University, United States of America

## Abstract

Environmental conditions early in life can affect an organism’s phenotype at adulthood, which may be tuned to perform optimally in conditions that mimic those experienced during development (Environmental Matching hypothesis), or may be generally superior when conditions during development were of higher quality (Silver Spoon hypothesis). Here, we tested these hypotheses by examining how diet during development interacted with diet during adulthood to affect adult sexually selected ornamentation and immune function in male mallard ducks (*Anas platyrhynchos*). Mallards have yellow, carotenoid-pigmented beaks that are used in mate choice, and the degree of beak coloration has been linked to adult immune function. Using a 2×2 factorial experimental design, we reared mallards on diets containing either low or high levels of carotenoids (nutrients that cannot be synthesized *de novo*) throughout the period of growth, and then provided adults with one of these two diets while simultaneously quantifying beak coloration and response to a variety of immune challenges. We found that both developmental and adult carotenoid supplementation increased circulating carotenoid levels during dietary treatment, but that birds that received low-carotenoid diets during development maintained relatively higher circulating carotenoid levels during an adult immune challenge. Individuals that received low levels of carotenoids during development had larger phytohemagglutinin (PHA)-induced cutaneous immune responses at adulthood; however, dietary treatment during development and adulthood did not affect antibody response to a novel antigen, nitric oxide production, natural antibody levels, hemolytic capacity of the plasma, or beak coloration. However, beak coloration prior to immune challenges positively predicted PHA response, and strong PHA responses were correlated with losses in carotenoid-pigmented coloration. In sum, we did not find consistent support for either the Environmental Matching or Silver Spoon hypotheses. We then describe a new hypothesis that should be tested in future studies examining developmental plasticity.

## Introduction

Beyond its genes, an animal’s morphology, physiology, and behavior are products of both the past and current environment [1]. Developmental conditions – ranging from hormone levels [2] to diet quality [3] to parasite exposure [4] – can have especially long-lasting or irreversible impacts on adult phenotype, including both survival- [5] and reproduction- [2], [6] related traits. However, despite a plethora of research on developmental plasticity, the manner in which the developmental and adult environments interact to affect fitness is still poorly understood.

Development can be an expanded and critical period for animals; it is typically characterized by high rates of growth [7], mortality [8] and investment in mechanical structures [9], neurological connections [10], and physiological pathways [11] that will be necessary to survive and reproduce at adulthood. However, the degree of investment in such traits can vary in fitness value depending on the type of environment in which the traits developed and in which type that the animal resides later in life. Monaghan [12] reviewed several hypotheses outlining how low- or high-quality environments during development could interact with low- or high-quality environments during adulthood to maximize the fitness value of a given phenotype. The two most prominent hypotheses are the Environmental Matching and the Silver Spoon hypotheses, while two other unnamed hypotheses incorporate elements of each (see [12] for further details). As the name suggests, the Environmental Matching hypothesis predicts that individuals that experience similar conditions (regardless of the quality of those conditions) during both development and adulthood will have a relatively higher fitness than those that experience mismatched conditions. Alternatively, the Silver Spoon hypothesis predicts that individuals exposed to superior conditions during development and/or adulthood have an increased fitness relative to those that experience poor conditions at any point. More generally, these two hypotheses differ in a fundamental way; the Environmental Matching hypothesis predicts that early- and late-life conditions interact to affect adult fitness, while the Silver Spoon hypothesis does not predict interactive effects.

Support for the Environmental Matching, the Silver Spoon, and the other hypotheses has been mixed. Predictions of the Environmental Matching hypothesis have been frequently supported in studies on humans (e.g., metabolism [13]). In contrast, many manipulations of diet quality or quantity [14]–[17], parasite exposure [14], or stress hormone (e.g., corticosterone) exposure [18] at both the developmental and adult stages in other organisms (e.g., butterflies, *Bicyclus anynana* [16]; zebra finches, *Taeniopygia guttata* [14]) have yielded support for the Silver Spoon hypothesis. In some cases, phenotypic effects of conditions during development do not persist beyond development. For example, manipulation of diet during development can be detrimental in the short term, but full compensation is possible given substantial time during adulthood [19]. Here, we propose to test the Environmental Matching and Silver Spoon models of developmental plasticity with respect to traits that are related to both survival and reproduction.

One strength of the studies cited above is the diverse effects of their manipulations on adult phenotype. Differences in food amount [16], [17], [19] or protein to carbohydrate ratio [14], [15] during development can affect an organism’s ability to express colorful ornaments [20], respond to an immune challenge [21], survive [22], and invest in reproduction [23]. While such broad manipulations of diet quality or quantity can be ecologically relevant and may be likely to affect multiple aspect of phenotype, some micronutrients also have myriad effects on developmental or adult phenotype, and these effects can be more tightly linked to specific physiological processes or phenotypic traits. For example, carotenoids are a group of molecules that are produced by photosynthetic organisms and that vertebrates must acquire from their diet [24]. These molecules act as pigments and are responsible for the red, orange, or yellow coloration of many fish [25], lizard [26], and avian [24] ornaments. These pigments can also support immune function [27]–[29] and may have antioxidant properties in biological systems [29], [30] but see [31], [32]. Correlational studies in adult humans and other animals have linked carotenoid levels to antibody production both *in* *vitro* [32] and *in* *vivo* [33], to the strength of the oxidative burst [34], and to the strength of the cutaneous immune response to phytohemagglutinin (PHA; [29]).

Carotenoid access during development also has a demonstrable impact on multiple facets of subsequent phenotype. Carotenoid access early in life can affect plumage coloration [35], behavior [36], and physiological assimilation of carotenoids [6] during later stages of development. Moreover, presence of carotenoids in the egg yolk of developing embryos affects their ability as neonates (4-week-old chicks; *Gallus gallus domesticus*; [37]) to incorporate dietary carotenoids into tissue mid-way through development. Therefore, a single class of molecules (carotenoids) affects traits related to both survival and reproduction at multiple life stages, and is thus ideal for assessing the interactive effects of past versus current environment on fitness-related traits.

To investigate these effects, we tested how dietary access to carotenoids during development and/or adulthood affected immune function and carotenoid-based ornament expression in male mallard ducks (*Anas platyrhynchos*). Males possess a yellow, carotenoid-pigmented beak [38], and this trait honestly reveals several aspects of current quality – such as sperm performance [33] and immune function [39] – to females that prefer yellower-beaked males as mates [40], [41]. Because the mechanisms of carotenoid physiology differ between ornamental coloration (deposition) and immune function (chemical reactions; [42]), we hypothesized that adult pigmentation and immune function would depend upon developmental and adult conditions differently. More explicitly, we hypothesized that the Environmental Matching hypothesis would apply to adult immune function and circulating carotenoid physiology, as individuals that did not under- or overexpress mechanisms of carotenoid storage or transport (e.g., lipoprotein expression to mobilize carotenoids; [43]) early in life would demonstrate a relatively superior immune response later in life by being able to mobilize and circulate appropriate levels of carotenoids. In accordance with the Environmental Matching hypothesis, we predicted that individuals receiving similar levels of carotenoids during both development and adulthood would maintain constant levels of circulating carotenoids throughout an adult immune challenge, have greater cutaneous immune response to phytohemagglutinin (PHA; [44]), and have a greater humoral response to a novel antigen. While a stronger immune response is not universally indicative of increased fitness [45], these particular metrics have been linked to increased nestling recruitment in songbirds ([46]–[48], but see [49]) and to more ornamented mallard drakes [33]. Separately, we hypothesized that variation in beak coloration would fit the predictions of the Silver Spoon hypothesis, as individuals with increased access to carotenoids during development would be more likely to store and subsequently deposit carotenoids in the integument, resulting in more yellow, ornamented beaks [38]. In accordance with the Silver Spoon hypothesis, we predicted additive effects of higher carotenoid diets during development and adulthood on carotenoid-rich beak coloration. Individuals receiving carotenoid-rich diets early in life may be able to store carotenoids (e.g., carotenoid storage in the liver; [50]) for subsequent ornamentation, or have an increased assimilation ability during adulthood [6], while adults receiving carotenoid-rich diets would be able to produce and maintain beak coloration because of their higher current intake and thus available pool of pigments to utilize. Finally, while we predicted that investigation of these variables would be more likely to be explained by either the Environmental Matching or the Silver Spoon hypotheses, our data permit us to evaluate both hypotheses simultaneously for all variables.

## Methods

### Experimental Protocol and Blood Collection

We acquired 42 one-day-old male ducklings from Metzer Farms (Gonzales, CA) in December 2010 and housed them as we have previously [51], [52]. Briefly, ducklings were reared indoors in randomly selected groups of four ducklings per cage (60×60×60 cm) until they were 21 days old, then in randomly selected groups of two per cage until 52 days old, at which point all birds were moved outside and individually housed to allow for normal sexual maturation [51], [53]. Light:dark regime was 13L:11D while ducklings were housed indoors, and followed the natural photoperiod thereafter (11.5L:12.5D at 52 days old to 14L:10D at 20 weeks old).

Individuals were randomly assigned to one of four treatment groups that varied in dietary carotenoid content. While the amount of carotenoids in yolk can affect post-hatch carotenoid physiology in birds [37], all ducklings circulated similar levels of carotenoids 2 days post-hatch (see results) and likely did not differ significantly across treatment groups in yolk carotenoid levels. Individuals were placed on either LOW or HIGH carotenoid diets from 2 to 49 days old (DEV period), which encompasses the entire period of growth [53]. To prepare diets, food (Mazuri Waterfowl Starter; Richmond, IN, USA) was mixed with sunflower oil that contained ORO-GLO dry pigmenter (2% carotenoids by mass, predominately lutein; Kemin AgriFoods North America, Inc., Des Moines, Iowa, USA). Treatment levels were based on pilot work that yielded the formulation of diets that contain 3 µg/g (LOW) and 25 µg/g (HIGH) of carotenoids. These amounts represent the first and third quartiles of carotenoid concentration found in wild duckling diets [54], of which the dominant carotenoid is lutein [54]. At 7 weeks of age, all individuals were transitioned from Waterfowl Starter to Waterfowl Maintenance (Mazuri) with no supplemental carotenoids added. During the DEV period, we measured body mass to the nearest g and tarsus length to the nearest 0.1 mm on a weekly basis. We also collected approximately 200 µl of whole blood in heparinized capillary tubes from ducklings when they were 2 days old, prior to initiation of dietary manipulation, and 300 µl when they were 49 days old, prior to cessation of DEV dietary treatment. Blood was stored on ice for several hrs and then centrifuged for 3 min at 10,000 rpm. We then aliquoted plasma into separate microtubes for carotenoid and hemagglutination/hemolysis assays (see below), and stored the plasma at −80°C until analysis.

When individuals were 119 days old (17 weeks old, age at which males have acquired breeding plumage and display courtship behavior; [7]), we measured body mass and tarsus length, and collected blood samples as above. Half of each original treatment group was then placed on either LOW or HIGH carotenoid diets that were formulated in the same manner as above, except that we used Waterfowl Maintenance chow. Mallards remained on this diet for the remainder of the experiment (20 weeks old), and hereafter we refer to this period as the ADULT period. This 2×2 design yielded 4 groups: LOW during DEV and ADULT (LL; *N* = 11), LOW during DEV and HIGH during ADULT (LH; *N* = 10), HIGH during DEV and LOW during ADULT (HL; *N* = 10), and HIGH during DEV and ADULT (HH; *N* = 11).

### Immune Assessment

**Figure 1:**
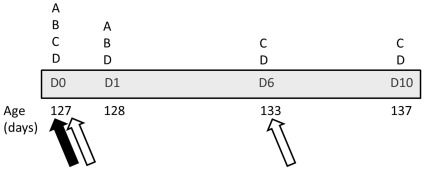
Timeline of adult immune assessment. Blood samples were collected, and beak color and mass were measured, at all four points (D0, D1, D6, and D10; see text for details). The letter “A” denotes when wing web thickness was measured, “B” denotes when NO level was measured, “C” denotes when KLH-specific antibody titer was measured, and “D” denotes when circulating carotenoid titer was measured. The filled arrow indicates when PHA was injected, and the open arrows indicate when KLH was administered.

One week into the ADULT period (to allow enough time for dietary treatment to be reflected in the individual’s physiology; see Results), we issued a series of immune challenges to assess each individual’s immune function (Figure 1). We have described these challenges in detail elsewhere [52], [55], so here we briefly review our methodologies.

On the first day of the adult immune assessment period (D0; Figure 1), we measured the thickness of the patagium (wing-web) in duplicate and then injected 0.1 mg of PHA (Sigma L8754) suspended in 0.1 mL of sterile phosphate buffered saline (PBS; Fisher BP399). Twenty-four hrs later (D1; mean: 23 hrs, 54 min; s.d.  = 16.4 min), we again measured the thickness of the wing web in duplicate. Measurements were significantly repeatable within each day (D0: R = 0.95; D1: R = 0.98; [56]), and we calculated swelling response as the difference between average thickness on D1 minus D0. A larger swelling response is associated with a more robust cutaneous immune response [44], [57].

Also on D0 (Figure 1), we administered an emulsion of 250 µl of Complete Freund’s Adjuvant (CFA; Difco Laboratories, Detroit, MI) and 250 µg of keyhole limpet hemocyanin (KLH; Sigma H7017) suspended in 250 µl of sterile ddH_2_O. Six days later (D6), we administered a booster injection of an emulsion of 250 µl of Incomplete Freund’s Adjuvant (IFA; Sigma F5506) and 250 µg of KLH suspended in 250 µl of sterile ddH_2_O. By collecting blood on D0, D6, and D10 (four days after D6), we were able to quantify humoral immune response to this novel antigen. Specifically, we used an ELISA [53], [55] to quantify KLH-specific antibody production, allowing us to measure primary (D6 minus D0) and secondary (D10 minus D0) humoral response. Because all individuals received both PHA and KLH challenges simultaneously, it is possible that our findings may have differed if individuals were required to only respond to a single challenge. However, due to the importance of assessing multiple aspects of immunity [58], we elected to perform both simultaneously, eliminating order effects that would have reduced our statistical power had we performed these challenges in sequence.

Both PHA [59] and CFA [60] induce systemic increases in nitric oxide (NO) levels, which is a marker of the oxidative burst in the immune response. Following [59], we quantified NO production in response to these immunostimulants on both D0 and D1. We deproteinized 15 µl of plasma [55] and followed previously established protocols [59] to quantify circulating NO levels on both D0 and D1 using a Greiss reaction, which involved measuring the absorbance of the final solution at 540 nm. NO response was calculated as the difference between D1 and D0. Absorbance values below the negative blank were assigned a value of 0, and the standard curve had an R^2^ = 0.994.

We used a hemolysis-hemagglutination assay [61] to assess immune function throughout development and adulthood without affecting immmunodevelopment. Natural antibodies (NAbs) are immunoglobulins (predominately IgM; [61]) that are formed without prior antigen exposure, and their presence can result in the clumping of foreign particles, including red blood cells (hemagglutination). NAbs also interact with complement to lyse foreign cells (e.g., red blood cells; [61]). Therefore, to assess both hemolysis and hemagglutination, we followed the protocol of [61] with several modifications. Using plasma samples collected at the beginning and end of the DEV (2 and 49 days old) and ADULT (119 and 137 days old) periods, we serially diluted 20 µl of plasma in PBS along a row of a 96-well plate, with the final column containing only PBS (negative control). We then added 20 µl of heparinized whole sheep blood (Hemostat Laboratories, Dixon, CA; SBH050) that was diluted 1∶100 in PBS to each well. We then incubated parafilm-covered plates at 42°C, which approximates the body temperature of mallard ducks [62], for 90 min. We then tilted the plates for 20 min at room temperature and scanned them using a flat-bed scanner (Hewlett-Packard Co., ScanJet 3670) at 600 dots per inch to measure hemagglutination. We then left the plates flat at room temperature for 70 min and rescanned for hemolysis (see [61] for scoring procedures).

### Carotenoid Titer and Coloration Assessment

We analyzed plasma carotenoid content using a hexane:methyl tert-butyl ether extraction method and high-performance liquid chromatography [63]. We quantified total carotenoid titer (predominately lutein and zeaxanthin; [55]) for all individuals at the beginning and end of DEV, the beginning of ADULT, and on D0, D1, D6, and D10 of the adult immune assessment period.

Ornamental beak coloration in mallards begins to develop by 10 weeks of age [7] and is completed in all birds by 16 weeks (MWB, pers. obs). On D0, D1, D6, and D10, we measured carotenoid-based beak coloration of adults from λ = 300–700 nm using an Ocean Optics (Dunedin, FL, USA) USB2000 spectrophotometer with a PX-2 pulsed xenon light source. *Sensu* [55], we measured a 1 cm band of the right dorso-lateral surface of the beak between the nares and the beak tip and binned measurements into 1 nm increments using CLRfiles (CLR version 1.05; [64]). We then used CLRvars (CLR version 1.05; [64]) to calculate the brightness (B1: total light reflectance), saturation (S1B: proportion of reflectance between 400–510 nm, the peak absorption area of many carotenoids, e.g., [54]), and hue (H4b: arctan of the reflectance within different regions of the light spectrum) scores that are most closely correlated with carotenoid content in the mallard beak (B1, negatively related to carotenoid content; S1B, negatively related to carotenoid content; H4b, positively related to carotenoid content; [38]).

### Statistics

Several of the variables that we quantified were not normally distributed. These variables (KLH primary response, NO levels on D0, and NO levels on D1) were log-transformed to achieve normality. However, hemolysis and hemagglutination could not be transformed to achieve normality, and the residuals from the described statistical models were also not normally distributed, so we also performed non-parametric tests for comparison (see below). For multivariate analysis of variance (MANOVA) tests, if the Greenhouse-Geisser (G-G) Epsilon was <0.7, we interpreted the G-G adjusted *P*-value. All post-hoc tests utilized Least Squares Means comparisons.

To test if circulating carotenoid titer, body mass, tarsus length, hemolysis, and hemagglutination differed as a function of treatment over time, we ran repeated-measures ANOVAs, using all 7 carotenoid data points (pre- and post-DEV, pre-ADULT, and D0, D1, D6, and D10), all 13 body mass points (8 times during DEV plus all 5 ADULT-stage measurements), all 9 tarsus length data points (8 times during DEV plus pre-ADULT), and the four hemolysis and hemagglutination points (pre- and post-DEV, pre-ADULT and D10). Because hemolysis and hemagglutination values could not be transformed to achieve normality, we also used non-parametric Friedman’s tests to see if there were differences between treatments within each age class, and within each treatment group as a function of age.

We tested how treatment during DEV, ADULT, and their interaction affected PHA-induced swelling, NO response, primary and secondary KLH response, and beak brightness, hue, and saturation by running separate ANOVAs for each dependent variable, with treatment during DEV, ADULT, and their interaction as independent factors. To test if adult beak color (at D0) predicted immune response, we performed simple linear regressions with beak saturation, hue, or brightness as the independent variable and PHA-induced swelling, NO response, primary and secondary KLH response, D10 hemolysis, or D10 hemagglutination as dependent variables. Lastly, to test how treatment and immune response may have combined to change adult carotenoid status or beak color, we performed a series of either ANOVA or analysis of covariance (ANCOVA) models. Specifically, we used ANOVAs to test if the change in circulating carotenoid titer over the first 1, 6, or 10 days of the adult immune assessment differed as a function of treatment. One individual circulated very high levels of carotenoids on the final day of the adult immune challenge (D10), resulting in a non-normal distribution of the data and associated residuals. Analyses with this individual excluded resulted in normally distributed data and residuals, and the statistics were qualitatively similar to those with the individual included. Therefore, because we had no *a priori* reason to exclude this individual, we present results with his data included. We also used ANCOVAs to test if change in beak color (hue, brightness, or saturation) was a function of treatment, using degree of immune response (e.g., PHA-induced swelling) over the same time period as a covariate.

## Results

### Effects of Diet Treatment on Developmental Metrics

**Figure 2:**
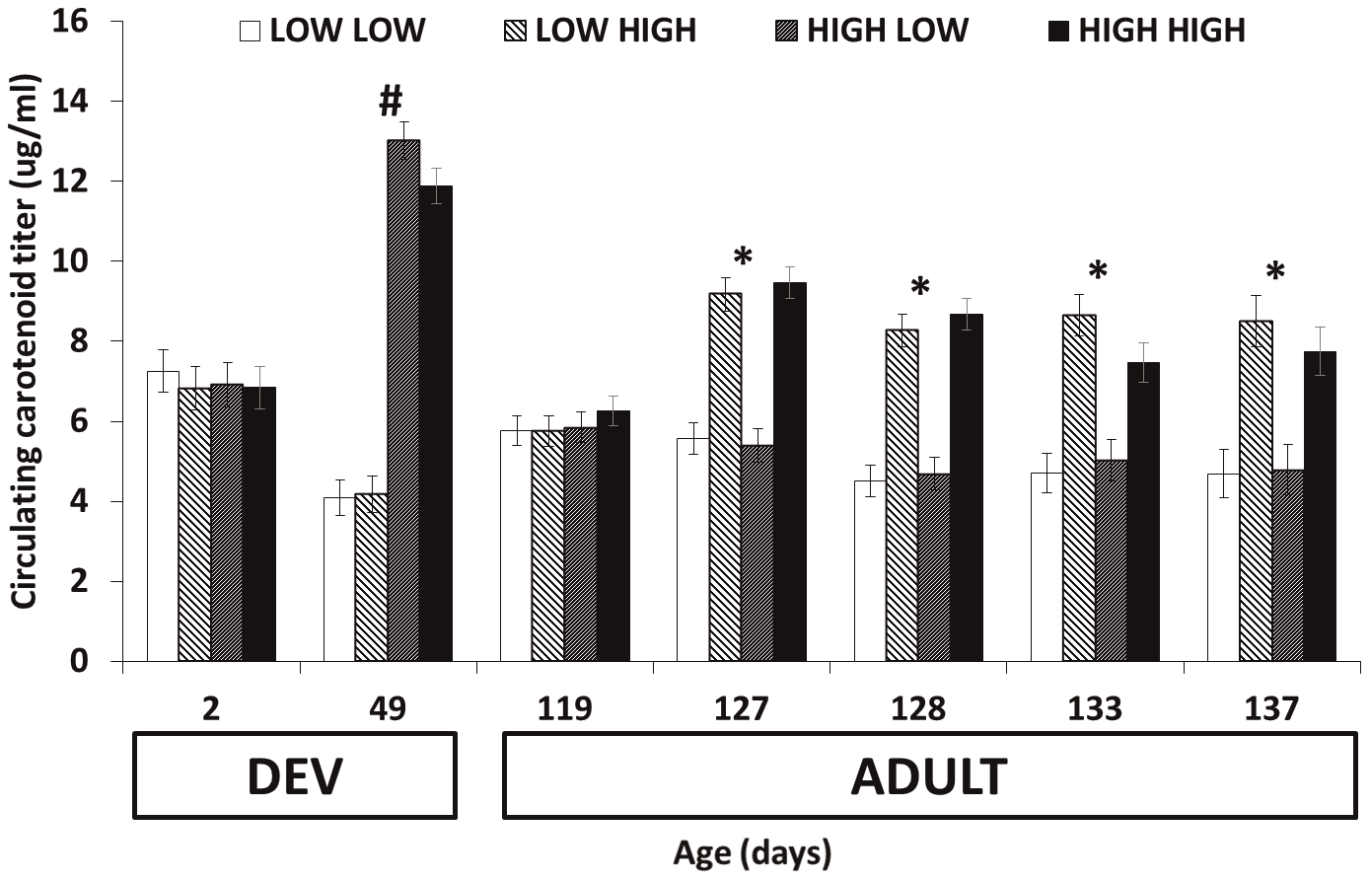
Circulating carotenoid titer as a function of age. Significant differences (*P*<0.05) within developmental treatment are denoted by #, and within adult treatment by *. Each treatment group is labeled based on access to LOW or HIGH levels of carotenoids during development (2–49 days old; first word in legend) and LOW or HIGH levels at adulthood (119–137 days old; second word in legend). HIGH levels of carotenoids during the first 7 weeks of development increased circulating carotenoid titer by the end of development, but these differences disappeared by the beginning of the adult stage. HIGH levels during adulthood increased circulating carotenoid titer within one week, and levels remained relatively higher throughout the remainder of the study.

Dietary carotenoid treatment during both DEV and ADULT affected circulating carotenoid levels (both F_1,38_>14.14, *P*<0.0006), with dietary treatment during both DEV (*F*_6,228_ = 63.33, *P*<0.0001) and ADULT (*F*_6,228_ = 26.17, *P*<0.0001) interacting with age. There were no effects of dietary treatment, age group, or their interaction on circulating carotenoid levels prior to DEV treatment (all *F*_1,38_<0.22, all *P*>0.6), but HL and HH birds circulated higher levels than LL and LH birds by the end of the DEV period (*F*_1,38_ = 334.55, *P*<0.0001). Just prior to the ADULT period, there were no differences in circulating carotenoid levels among treatment groups (all *F*_1,38_<0.59, all *P*>0.4), but at all subsequent time points LH and HH birds circulated higher levels of carotenoids than LL and HL birds (all *F*_1,38_<30.18, all *P*<0.0001). There were no other significant interactions (all *P*>0.33; Figure 2).

Dietary treatment during DEV (*F*_1,38_ = 0.41, *P* = 0.53), ADULT (*F*_1,38_ = 0.41, *P* = 0.53), and their interaction (*F*_1,38_ = 0.05, *P* = 0.83) did not affect body mass, nor did any of these main effects interact with age (all *F*_12,456_<0.58, all *P*>0.6). Similarly, there was no effect of dietary treatment during DEV (*F*_1,38_ = 0.79, *P* = 0.38), ADULT (*F*_1,38_ = 2.98, *P* = 0.093), or their interaction (*F*_1,38_ = 0.61, *P* = 0.44) on tarsus length, nor did any of these main effects interact with age (all *F*_8,304_<1.90, all *P*>0.13) to affect tarsus length.

According to rmANOVAs that did not have normally distributed residuals (see above), both agglutination (*F*_3,114_ = 146.43, *P*<0.0001) and lysis (*F*_3,114_ = 163.05, *P*<0.0001) increased with age, but there was no effect of either dietary treatment (all *F*_1,38_<2.55, all *P*>0.12) or an interaction with treatment and age (all *F*_3,114_<0.85, all *P*>0.42) for either metric. When analyzing these data using non-parametric Friedman’s tests, the results were statistically similar, with effects of age within all treatment groups on both agglutination (χ^2^ = 46.3, d.f.  = 3, *P*<0.0001) and lysis (χ^2^ = 43.3, d.f.  = 3, *P*<0.0001), and no effects of treatment on agglutination or lysis scores at any age (all χ^2^<2.91, d.f.  = 1, all *P*>0.088).

### Effects of Dietary Treatment on Adult-stage Metrics

**Figure 3:**
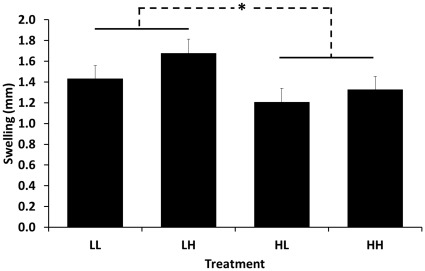
PHA-induced wing web swelling as a function of dietary treatment. The first letter of the treatment denotes whether individuals received LOW (L) or HIGH (H) levels of carotenoids in the diet, while the second letter denotes the diet received at the adult stage, concurrent with the PHA challenge. Those that received LOW diets during development (hatch –7 weeks old) had larger swellings (*; *P*<0.05) than those that received HIGH diets.

Birds that received the LOW diet during DEV had greater PHA-induced swellings than HL and HH birds (*F*_1,38_ = 4.84, *P* = 0.034), but there was no effect of ADULT dietary treatment or the interaction between diet during DEV and ADULT (both *F*_1,38_<1.99, both *P*>0.16; Figure 3) on PHA-induced swelling. Dietary treatment during DEV, ADULT, and their interaction did not affect primary KLH response (all *F*_1,38_<0.32, all *P*>0.58), secondary KLH response (all *F*_1,38_<0.34, all *P*>0.56), or NO response (all *F*_1,38_<2.30, all *P*>0.14). Additionally, adult beak color assessed immediately prior to adult immune assessment did not differ by dietary treatment during DEV, ADULT, or their interaction (brightness: all *F*_1,38_<2.88, all *P*>0.098; saturation: all *F*_1,38_<0.46, all *P*>0.5; hue: all *F*_1,38_<0.67, all *P*>0.4).

### Adult Beak Color Predicting Adult Immune Function

**Figure 4:**
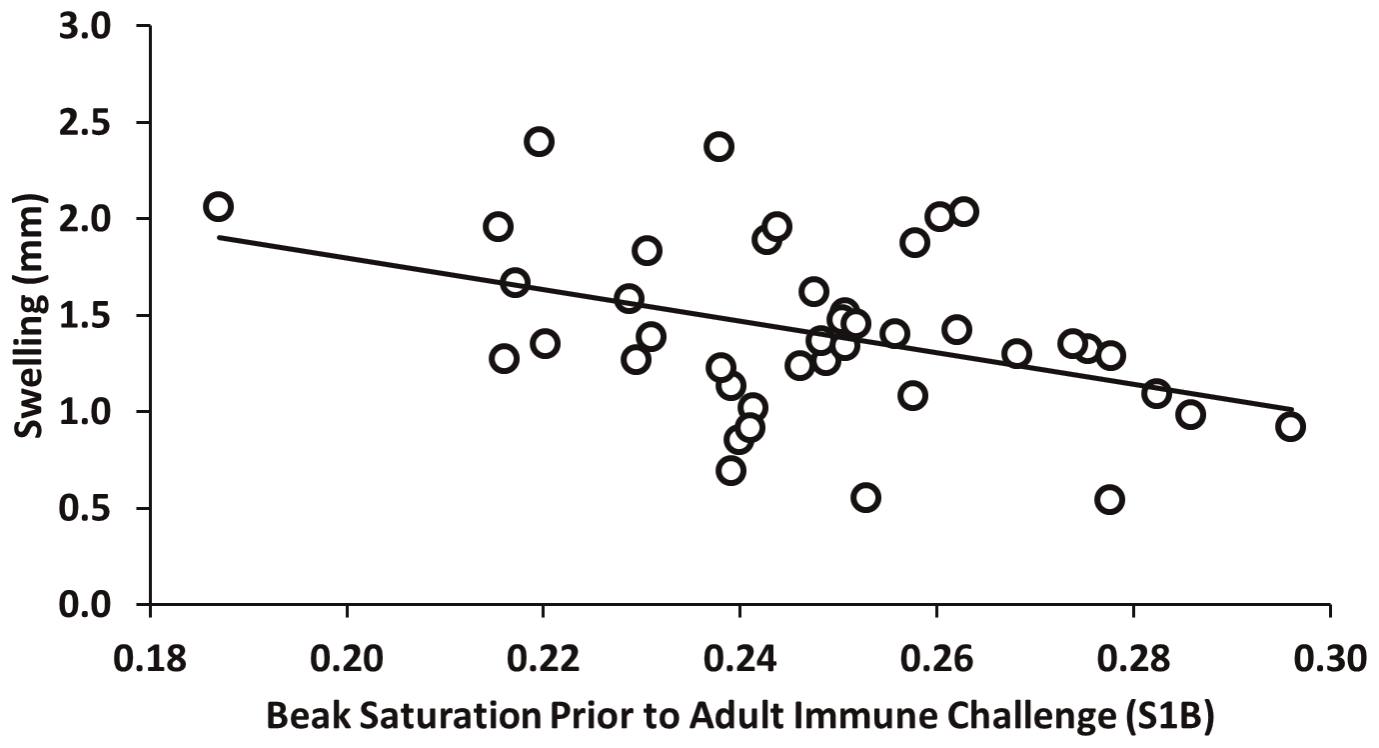
Beak color predicting PHA-induced wing web swelling. Individuals with larger S1B values, or saturation of the blue component of the spectrum [64], had smaller PHA-induced swelling responses. Because S1B is inversely correlated with carotenoid content of mallard beaks [38], males with more carotenoid-rich beaks had larger PHA-induced swelling responses.

Beak saturation was related to PHA-induced swelling, with a greater PHA response associated with a more carotenoid-rich beak (lower S1B, *F*_1,40_ = 7.76, *P* = 0.0081; Figure 4). Beak saturation did not predict primary or secondary KLH response, NO response, or hemagglutination or hemolysis on D10 (all *F*_1,40_<1.54, all *P*>0.22), and neither beak brightness nor hue predicted any immune metric (all *F*_1,40_<2.25, all *P*>0.14).

### Effects of Dietary Carotenoid Treatment and Immune Response on Change in Adult Phenotype During the Adult Immune Assessment Period

**Figure 5:**
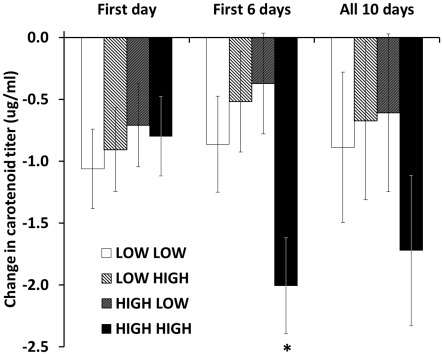
Change in carotenoid titer throughout the adult immune assessment period. Groups are described based on whether they received LOW or HIGH levels of carotenoids during development (2–49 days old; first word in legend) and LOW or HIGH levels at adulthood (119–137 days old; second word in legend). HIGH HIGH birds showed a larger decrease in circulating carotenoid levels over the first six days of the adult immune assessment than all other groups (*; all *P*<0.05).

Neither change (all *F*_1,38_<0.49, all *P*>0.49) nor percent change (all *F*_1,38_<3.01, all *P*>0.091) in carotenoid levels during the first 24 hours of the immune challenge (D0 to D1) differed by dietary treatment during DEV, ADULT, or their interaction. However, there was a significant interaction effect of diet during DEV and ADULT on change in carotenoid levels over the first six days of the KLH immune challenge period (D0 to D6; *F*_1,38_ = 6.18, *P* = 0.017), with HH birds decreasing circulating carotenoid levels more than LL, LH, and HL birds (all *P*<0.044; Figure 5). The result was similar if analyzed as a function of percent change (DEV*ADULT; *F*_1,38_ = 5.35, *P* = 0.026), with HH birds decreasing circulating carotenoid levels by a greater percentage than both LH and HL (both *P*<0.05), but not LL (*P* = 0.35) birds. There was no significant effect of dietary treatment during DEV, ADULT, or their interaction on change (all *F*_1,38_<1.14, all *P*>0.29) or percent change (all *F*_1,38_<1.44, all *P*>0.24) in circulating carotenoid levels over the course of the 10-day adult immune challenge.

**Figure 6:**
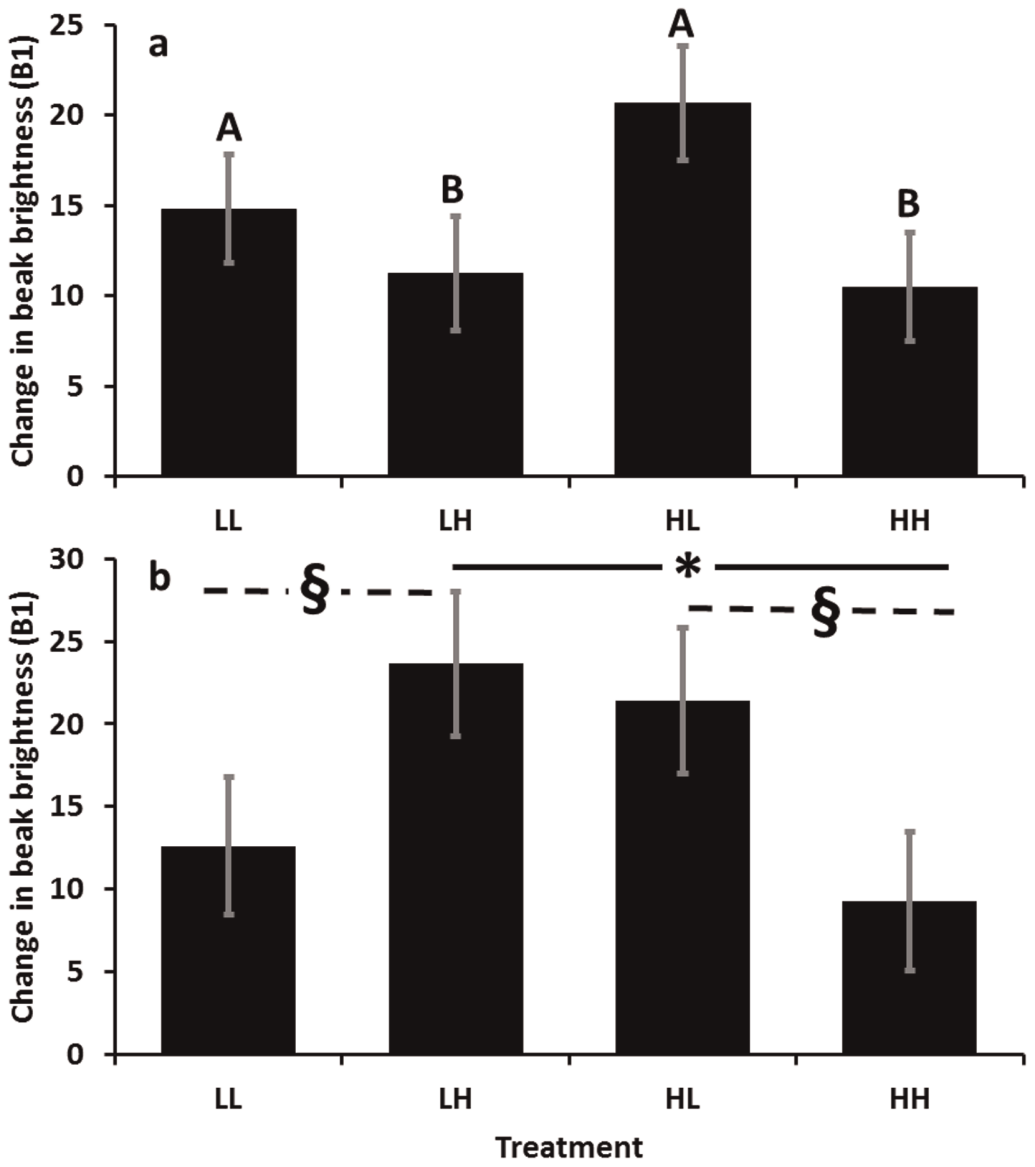
Change in beak brightness over the first (a) 6 and (b) 10 days of the adult immune assessment as a function of treatment. The first letter of the treatment label denotes whether individuals received LOW (L) or HIGH (H) levels of carotenoids in the diet, while the second letter denotes the diet received at the adult stage. An increase in brightness is associated with a loss of carotenoid pigment [38]. Therefore, over the first six days of the adult immune assessment, individuals receiving LOW diets (A) had beaks that became less carotenoid-rich (i.e., increased in brightness) –relative to those receiving HIGH diets (B; *P*<0.05). Over the full 10 days of immune assessment, however, LH bird beaks became more carotenoid-depleted than HH bird beaks (*; *P*<0.05), while similar trends existed between LH and LL birds, and HL and HH birds (§; 0.05<*P*<0.08).

Beak saturation changed significantly in proportion to PHA-induced swelling response (*F*_1,37_ = 6.32, *P* = 0.016), with a greater response associated with a loss of carotenoid-based coloration, but there was no effect of diet during DEV, ADULT, or their interaction on beak saturation (all *F*_1,37_<0.42, all *P*>0.5). Beak brightness changed over the course of the first six days of adult immune assessment as a function of ADULT diet (*F*_1,37_ = 4.96, *P* = 0.032), with LL and HL birds decreasing in carotenoid-based coloration more than LH and HH birds (Figure 6a). Change in beak brightness over the course of the 10-day immune assessment differed by the interaction between DEV and ADULT (*F*_1,37_ = 7.29, *P* = 0.010), with LH birds decreasing in carotenoid-based coloration more than HH birds (*P*  = 0.0239), and non-significantly decreasing more than LL birds (*P* = 0.076). There was also a non-significant trend for HL birds to decrease in carotenoid based coloration more than HH (*P* = 0.054) birds (Figure 6b). All other effects for change in saturation and brightness were not significant (all *F*_1,37_<3.12, all *P*>0.086), and there were no significant relationships between change in beak hue and treatment or degree of immune response (all *F*_1,37_<2.58, all *P*>0.12).

## Discussion

We found that differential carotenoid intake during development affected adult immune response and interacted with adult carotenoid access to affect circulating carotenoid levels and maintenance of a carotenoid-pigmented ornament during an immune challenge in mallard ducks. We hypothesized that carotenoid-associated immune responses and carotenoid physiology would follow predictions for the Environmental Matching hypothesis, while carotenoid-dependent ornamentation would follow predictions of the Silver Spoon hypothesis; however, we found no support for such relationships. Instead, our results demonstrate that ornament maintenance most closely follows the predictions of Environmental Matching. More surprisingly, and in line with none of Monaghan’s developmental plasticity hypotheses [12], we found that individuals exposed to HIGH levels of carotenoids during development either had similar (e.g., KLH-induced antibody production) or reduced (e.g., PHA-induced swelling) levels of adult immune function, depending on the specific immune metric, relative to LL and LH birds. Similarly, HH had larger decreases in circulating carotenoid levels during those immune challenges without benefiting from an increase in immune function. Taken together, these findings suggest that HIGH levels of carotenoids during development may serve no inherent benefit at adulthood, and may actually be detrimental in select environments.

Individuals that received LOW diets during development had a greater PHA-induced swelling at adulthood, regardless of adult carotenoid treatment. A greater PHA-induced swelling has generally been associated with birds in better condition [65], [66], but see [67]. Our findings, therefore, are not consistent with the Environmental Matching hypothesis, and stand in direct opposition to the Silver Spoon hypothesis, as lower levels of carotenoids are presumed to be indicative of poorer conditions. However, although only occasionally discussed, high levels of carotenoids may actually be detrimental [68]. Previous work with American goldfinches (*Carduelis tristis*) showed that high levels of carotenoids negatively affected flight performance [69], but in that study, there was a 100-fold increase in carotenoid access between low- and high-carotenoid treatments. Our treatment reflected approximately an 8-fold increase, and circulating carotenoid levels in HIGH birds were similar to those found in wild ducklings [54], so it is unlikely that levels of carotenoid supplementation were pharmacological. Therefore, our data demonstrate that individuals that have access to low, but ecologically relevant, levels of carotenoids during development will have an increased cutaneous immune response at adulthood, although the precise mechanism for this result is unknown. PHA-induced swelling reflects multiple components of immune activity [57], and thus may be an important immune metric. However, we tested other aspects of immune function, including markers of humoral (primary and secondary anti-KLH antibody production) and innate (NO response, hemagglutination and hemolysis capacity) immune function, and did not find any other treatment differences. Therefore, at least in mallards, access to carotenoids during development seems to affect only one component of the immune system, supporting the expanding view that, while carotenoids do increase immune function in several cases [28], [29], they do not necessarily increase response in all axes of the immune system (PHA-induced swelling: [31]; antibody production: [70]; markers of innate immunity: [71]).

Interestingly, dietary treatment during DEV and ADULT did not affect adult measures of beak coloration. While access to carotenoids over 10 weeks during adulthood affected adult drake beak coloration in a separate study [72], it is possible that, due to the length of time required for tissue turnover within the beak integument, these differences do not manifest within the first few weeks of supplementation, thus accounting for the lack of effect of 4-week carotenoid supplementation during the ADULT stage of our study. However, the lack of an effect due to DEV diet suggests little need for young male ducklings to consume carotenoids to ensure maximal expression of beak coloration later in life (but see below, [73]).

In accordance with previous studies [39], [54], we found that male beak color prior to an immune challenge was predictive of the degree of immune response and that beaks declined in carotenoid levels during the time course of the immune response. These findings are consistent with a role for mallard male beak coloration in honestly signaling current immune function to females. Similar short-term changes to carotenoid-based ornaments have previously been demonstrated in both mallards [53] and red grouse (*Lagopus lagopus scoticus*; [74]). Such findings highlight the potential functional consequences of ornament lability, and future work should test for a female’s ability to detect and make mate-choice decisions using intra-individual differences in coloration over time as a marker of current investment in immunity. In addition to the correlation between immune response and degree of color change described above, we found dietary treatment effects on change in beak coloration over the first 6 and 10 days of immune challenges. Over the first six days, adult males showed a greater decrease in carotenoid-based coloration if they were currently receiving LOW diets, suggesting that, while several weeks of prior access to carotenoids may not directly affect the absolute level of carotenoid-based coloration (see above), concurrent access may affect color change during an immune response. Over the full 10 days of adult immune assessment, however, we detected color changes that provide limited support for the Environmental Matching hypothesis; that is, individuals that experienced similar levels of carotenoids during both development and adulthood (LL and HH birds) tended not to decrease in carotenoid-based coloration as much as those that experienced dissimilar diets (LH and HL birds). Some of these relationships were not statistically significant, so these results should be viewed as preliminary findings that require further investigation. Work with red grouse [74], [75] and zebra finches (*Taeniopygia guttata*; [76]) has demonstrated several physiological mechanisms by which carotenoid-based ornamentation may be maintained (e.g., hormone-dependent changes in physiology), and future behavioral tests could provide a functional context to the relative importance of both lability in ornamentation and average level of ornament expression play inmate choice patterns.

Circulating carotenoid levels decreased throughout the adult immune assessment period in all groups. Both this finding and the fading of beak coloration during an immune challenge are consistent with the utilization of carotenoids during an immune response [77], although the lack of relationships between ADULT dietary treatment and most immune metrics, as well as the lack of a control, unchallenged group, does not allow us to experimentally support this position. However, we did detect treatment differences in the degree of change in circulating carotenoid levels, specifically that HH individuals decreased circulating carotenoid levels more than any other group over the first 6 days of the adult immune assessment. While HH individuals had higher initial levels relative to LL and HL (treatment effect of diet during ADULT), and thus may have been able to decrease levels relatively more without suffering any adverse effects, HH birds also decreased circulating carotenoid levels more than LH birds. This finding suggests that exposure to low levels of carotenoids during DEV resulted in LH birds’ having an increased ability to assimilate or mobilize carotenoids relative to HH birds during an immune challenge. Carotenoids are generally transported by lipoproteins [43], [78], and a potentially fruitful area of research may be to test how developmental access to carotenoids affects lipoprotein profiles in adults exposed to varying levels of carotenoids to discover a possible mechanism for the developmental plasticity of carotenoid physiology.

While our results did not support our predictions, we did uncover multiple examples of how dietary carotenoid content during development affected aspects of adult phenotype, either alone or in concert with carotenoid access during adulthood. Unexpectedly, we uncovered evidence that does not fit any of the traditional models of developmental plasticity [12]. Specifically, DEV LOW birds had a greater PHA-induced swelling at adulthood, and LH birds more effectively maintained circulating carotenoid levels over the first six days of an adult immune challenge than HH birds. Taken together, these findings point to an advantage at adulthood of experiencing low-quality (i.e., low-carotenoid) conditions during development, even in high-quality adult conditions. Thus, we propose that a fifth model of developmental plasticity be considered in future studies: the hard-knock life hypothesis. Specifically, we propose that, if the costs of responding to poor-developmental conditions are low enough, individuals may invest in physiological or neurological processes that provide an adaptive advantage in all adult conditions, provided that the individual survives the relatively poor developmental conditions (a requirement that was easily met while using captive birds).

There are three important predictions for this hypothesis. First, the costs (in general fitness units) to responding to the poor developmental condition must be smaller than the benefits at adulthood, or else the hypothesis is similar to the Silver Spoon hypothesis. Second, the costs during development must be large enough to decrease the expected lifetime fitness of the individual, most likely via a reduction in short-term survival probability, or selection would act on all individuals to invest in these changes. Third, for the subset of individuals that do survive, the resulting phenotypic changes should yield an increase in fitness in future adult environments. Data supporting this hypothesis have been found not only in this study, but in others as well, with neonatal food deprivation resulting in subsequent increased immune function in lizards (*Zootoca vivipara*; [79]), and developmental food restriction in the larval stage yielding adult butterflies (*Bicyclus anynana*) that coped better with forced flight [16]. Thus, in addition to testing the hypotheses set forth in Monaghan’s [12] work, future researchers should also test the hard-knock life hypothesis, especially for relatively small, but biologically relevant developmental perturbations, including moderately pathogenic immune challenges, micronutrient availability, or low levels of physiological stress.
